# Supplementary material for: Modeling glioblastoma heterogeneity as a dynamic network of cell states
Source: Mol Syst Biol. 2021 Sep 16;17(9):e10105. doi: 10.15252/msb.202010105 (PMC8444284; doi:10.15252/msb.202010105)
Supplement: Supplementary file 6 — Source Data for Figure 5 [file MSB-17-e10105-s004.zip › Figure5A_sourcedata/GSEA_3017/nosigdb_stateC.GseaPreranked.1622795002785/LARSSON_STATE_4.html]

Details for gene set LARSSON\_STATE\_4[GSEA]

|  || Dataset | state13017 |
| Phenotype | NoPhenotypeAvailable |
| Upregulated in class | na\_pos |
| GeneSet | LARSSON\_STATE\_4 |
| Enrichment Score (ES) | 0.7541505 |
| Normalized Enrichment Score (NES) | 5.1157794 |
| Nominal p-value | 0.0 |
| FDR q-value | 0.0 |
| FWER p-Value | 0.0 |
Table: GSEA Results Summary

  

Fig 1: Enrichment plot: LARSSON\_STATE\_4      
 Profile of the Running ES Score & Positions of GeneSet Members on the Rank Ordered List

  

| PROBE | GENE SYMBOL | GENE\_TITLE | RANK IN GENE LIST | RANK METRIC SCORE | RUNNING ES | CORE ENRICHMENT || 1 | ATAD2 |  |  | 7 | 0.649 | 0.0190 | Yes |
| 2 | HIST1H4C |  |  | 9 | 0.603 | 0.0468 | Yes |
| 3 | CENPU |  |  | 10 | 0.588 | 0.0756 | Yes |
| 4 | HIST1H1C |  |  | 11 | 0.583 | 0.1042 | Yes |
| 5 | HIST1H1D |  |  | 12 | 0.583 | 0.1328 | Yes |
| 6 | EZH2 |  |  | 14 | 0.574 | 0.1591 | Yes |
| 7 | CLSPN |  |  | 17 | 0.561 | 0.1830 | Yes |
| 8 | RRM2 |  |  | 19 | 0.542 | 0.2077 | Yes |
| 9 | HMGB2 |  |  | 20 | 0.534 | 0.2339 | Yes |
| 10 | CHAF1A |  |  | 27 | 0.494 | 0.2471 | Yes |
| 11 | PCLAF |  |  | 29 | 0.484 | 0.2690 | Yes |
| 12 | UBE2T |  |  | 31 | 0.475 | 0.2905 | Yes |
| 13 | HIST1H1B |  |  | 35 | 0.460 | 0.3076 | Yes |
| 14 | GMNN |  |  | 38 | 0.453 | 0.3261 | Yes |
| 15 | ORC6 |  |  | 39 | 0.450 | 0.3482 | Yes |
| 16 | ESCO2 |  |  | 43 | 0.440 | 0.3643 | Yes |
| 17 | UHRF1 |  |  | 45 | 0.436 | 0.3838 | Yes |
| 18 | RAD51AP1 |  |  | 46 | 0.435 | 0.4052 | Yes |
| 19 | PCNA |  |  | 48 | 0.435 | 0.4247 | Yes |
| 20 | TMPO |  |  | 49 | 0.432 | 0.4459 | Yes |
| 21 | HIST1H1E |  |  | 51 | 0.430 | 0.4652 | Yes |
| 22 | TOP2A |  |  | 52 | 0.426 | 0.4860 | Yes |
| 23 | BRCA1 |  |  | 54 | 0.410 | 0.5043 | Yes |
| 24 | DHFR |  |  | 55 | 0.405 | 0.5242 | Yes |
| 25 | TYMS |  |  | 60 | 0.390 | 0.5360 | Yes |
| 26 | RRM1 |  |  | 62 | 0.386 | 0.5531 | Yes |
| 27 | SMC2 |  |  | 63 | 0.386 | 0.5720 | Yes |
| 28 | E2F1 |  |  | 67 | 0.380 | 0.5852 | Yes |
| 29 | DEK |  |  | 74 | 0.373 | 0.5925 | Yes |
| 30 | CENPH |  |  | 77 | 0.366 | 0.6068 | Yes |
| 31 | SMC4 |  |  | 78 | 0.365 | 0.6247 | Yes |
| 32 | GINS2 |  |  | 83 | 0.360 | 0.6350 | Yes |
| 33 | STMN1 |  |  | 85 | 0.357 | 0.6507 | Yes |
| 34 | DUT |  |  | 87 | 0.356 | 0.6663 | Yes |
| 35 | MCM7 |  |  | 91 | 0.352 | 0.6781 | Yes |
| 36 | NUSAP1 |  |  | 100 | 0.340 | 0.6802 | Yes |
| 37 | MCM3 |  |  | 114 | 0.324 | 0.6723 | Yes |
| 38 | H2AFX |  |  | 115 | 0.322 | 0.6881 | Yes |
| 39 | HMGN2 |  |  | 126 | 0.312 | 0.6851 | Yes |
| 40 | FBXO5 |  |  | 130 | 0.308 | 0.6947 | Yes |
| 41 | KIF15 |  |  | 136 | 0.302 | 0.7004 | Yes |
| 42 | NCAPG |  |  | 137 | 0.301 | 0.7151 | Yes |
| 43 | CENPK |  |  | 139 | 0.299 | 0.7279 | Yes |
| 44 | LMNB1 |  |  | 142 | 0.293 | 0.7386 | Yes |
| 45 | ASF1B |  |  | 143 | 0.291 | 0.7529 | Yes |
| 46 | SIVA1 |  |  | 151 | 0.286 | 0.7542 | Yes |
| 47 | MYBL2 |  |  | 164 | 0.278 | 0.7458 | No |
| 48 | MAD2L1 |  |  | 174 | 0.270 | 0.7426 | No |
| 49 | MCM4 |  |  | 189 | 0.259 | 0.7297 | No |
| 50 | CDK1 |  |  | 191 | 0.255 | 0.7404 | No |
Table: GSEA details [plain text format]

  

Fig 2: LARSSON\_STATE\_4: Random ES distribution      
 Gene set null distribution of ES for **LARSSON\_STATE\_4**
